# Supplementary material for: Dynamics of Cellulose Degradation by Soil Microorganisms from Two Contrasting Soil Types
Source: Microorganisms. 2024 Aug 21;12(8):1728. doi: 10.3390/microorganisms12081728 (PMC11356995; doi:10.3390/microorganisms12081728)
Supplement: Supplementary file 1 [file microorganisms-12-01728-s001.zip › microorganisms-3132235-supplementary/Supplement_1.html]

Dynamics of cellulose degradation by soil microorganisms from two contrasting soil types


Code 

- Show All Code
- Hide All Code

# Dynamics of cellulose degradation by soil microorganisms from two contrasting soil types

#### Grigory Gladkov

#### 2024-07-15

```
library(tidyverse)
library(phyloseq)
library(igraph)
library(SpiecEasi)
```

```
psits.f <- readRDS("psits.f")
ps.f <- readRDS("ps.f")
```

## 0.1 16S basic EDA

```
plot_rich_reads_samlenames_lm(ps.f, group = "Repeat", label = "Repeat")
```

```
p1 <- plot_alpha_w_toc_2(ps.f, group="Repeat", metric="Observed")
p2 <- plot_alpha_w_toc_2(ps.f, group="Repeat", metric="Shannon")
p3 <- plot_alpha_w_toc_2(ps.f, group="Repeat", metric="InvSimpson")

p_alpha <- ggpubr::ggarrange(p1, p2, p3, nrow = 1)
p_alpha
```

```
amp <- phyloseq_to_ampvis2(ps.f)
amp
```

```
## ampvis2 object with 5 elements. 
## Summary of OTU table:
## [1]       35     2427   784466     7707    31969    22866 22413.31
## 
## Assigned taxonomy:
##      Kingdom       Phylum        Class        Order       Family        Genus 
##   2427(100%)   2427(100%) 2385(98.27%) 2231(91.92%) 1954(80.51%) 1379(56.82%) 
##      Species 
##   160(6.59%) 
## 
## Metadata variables: 6 
##  SampleID, Substrate, Repeat, Time, Respiration, Day
```

```
ampvis2::amp_heatmap(amp,
            tax_show = 22,
            group_by = "Day",
            facet_by = "Substrate",
            tax_aggregate = "Phylum",
            tax_class = "Proteobacteria",
            normalise=TRUE,
            showRemainingTaxa = TRUE)
```

```
ampvis2::amp_heatmap(amp,
            tax_show = 60,
            group_by = "Day",
            facet_by = "Substrate",
            tax_aggregate = "Genus",
            tax_add = "Phylum",
            normalise=TRUE,
            plot_values_size = 3, 
            showRemainingTaxa = TRUE)
```

```
ampvis2::amp_heatmap(amp %>% ampvis2::amp_filter_taxa("Bacteroidota", normalise = TRUE),
            tax_show = 40,
            group_by = "Day",
            facet_by = "Substrate",
            tax_aggregate = "Species",
            tax_add = "Genus",
            normalise=FALSE,
            plot_values_size = 3, 
            showRemainingTaxa = TRUE)
```

## 0.2 its2S basic EDA

Previosly some sample was removed, based on this picture(‘its2\_C14.4’)  
Isn’t shown here, because crean code should stay clean.

```
plot_rich_reads_samlenames_lm(psits.f, 'Repeat')
```

## 0.3 final beta`s plot

```
p.beta.16 <- beta_custom_norm_NMDS_elli_w(ps.f, Group = "Repeat", Color = "Substrate")
```

```
## Square root transformation
## Wisconsin double standardization
## Run 0 stress 0.1414392 
## Run 1 stress 0.1414393 
## ... Procrustes: rmse 0.0001039841  max resid 0.0005090306 
## ... Similar to previous best
## Run 2 stress 0.1414392 
## ... New best solution
## ... Procrustes: rmse 0.0001358634  max resid 0.0007061268 
## ... Similar to previous best
## Run 3 stress 0.1414393 
## ... Procrustes: rmse 0.0002067268  max resid 0.001028533 
## ... Similar to previous best
## Run 4 stress 0.1414392 
## ... New best solution
## ... Procrustes: rmse 3.896502e-05  max resid 0.0001980033 
## ... Similar to previous best
## Run 5 stress 0.1414394 
## ... Procrustes: rmse 0.0002258226  max resid 0.001135017 
## ... Similar to previous best
## Run 6 stress 0.1414394 
## ... Procrustes: rmse 0.0001964429  max resid 0.0009827264 
## ... Similar to previous best
## Run 7 stress 0.252504 
## Run 8 stress 0.1414392 
## ... Procrustes: rmse 2.018293e-05  max resid 0.000102256 
## ... Similar to previous best
## Run 9 stress 0.2096128 
## Run 10 stress 0.2369454 
## Run 11 stress 0.3956221 
## Run 12 stress 0.1414393 
## ... Procrustes: rmse 0.0001361324  max resid 0.0007107939 
## ... Similar to previous best
## Run 13 stress 0.1414392 
## ... Procrustes: rmse 3.50637e-05  max resid 0.00018067 
## ... Similar to previous best
## Run 14 stress 0.1414393 
## ... Procrustes: rmse 0.0001665753  max resid 0.0008499142 
## ... Similar to previous best
## Run 15 stress 0.1414393 
## ... Procrustes: rmse 0.0001602456  max resid 0.000841998 
## ... Similar to previous best
## Run 16 stress 0.1414392 
## ... Procrustes: rmse 8.376874e-05  max resid 0.0004249862 
## ... Similar to previous best
## Run 17 stress 0.2137143 
## Run 18 stress 0.1414392 
## ... Procrustes: rmse 0.0001250035  max resid 0.0006321025 
## ... Similar to previous best
## Run 19 stress 0.1414392 
## ... Procrustes: rmse 6.714066e-05  max resid 0.0003451903 
## ... Similar to previous best
## Run 20 stress 0.1414392 
## ... Procrustes: rmse 3.107439e-05  max resid 0.0001579937 
## ... Similar to previous best
## *** Best solution repeated 12 times
```

```
p.beta.its <- beta_custom_norm_NMDS_elli_w(psits.f, Group = "Repeat", Color = "Substrate")
```

```
## Square root transformation
## Wisconsin double standardization
## Run 0 stress 0.1844704 
## Run 1 stress 0.2491916 
## Run 2 stress 0.1837427 
## ... New best solution
## ... Procrustes: rmse 0.03782368  max resid 0.1504814 
## Run 3 stress 0.1837427 
## ... Procrustes: rmse 1.161844e-05  max resid 5.355889e-05 
## ... Similar to previous best
## Run 4 stress 0.2180571 
## Run 5 stress 0.1837427 
## ... Procrustes: rmse 1.228473e-05  max resid 4.614566e-05 
## ... Similar to previous best
## Run 6 stress 0.2397711 
## Run 7 stress 0.2407366 
## Run 8 stress 0.2545162 
## Run 9 stress 0.1844704 
## Run 10 stress 0.2366068 
## Run 11 stress 0.2307838 
## Run 12 stress 0.1844704 
## Run 13 stress 0.1844704 
## Run 14 stress 0.1837427 
## ... Procrustes: rmse 2.600436e-06  max resid 7.748832e-06 
## ... Similar to previous best
## Run 15 stress 0.1844704 
## Run 16 stress 0.1837427 
## ... New best solution
## ... Procrustes: rmse 2.588151e-06  max resid 9.389835e-06 
## ... Similar to previous best
## Run 17 stress 0.1837427 
## ... Procrustes: rmse 2.088062e-06  max resid 5.973571e-06 
## ... Similar to previous best
## Run 18 stress 0.2180571 
## Run 19 stress 0.2402092 
## Run 20 stress 0.1837427 
## ... New best solution
## ... Procrustes: rmse 3.007638e-06  max resid 9.064927e-06 
## ... Similar to previous best
## *** Best solution repeated 1 times
```

```
p.beta.16.mod <- p.beta.16 + 
  ggtitle('16S', subtitle = 'NMDS - Bray-Curtis dissimilarity') +
      scale_colour_viridis_d(option = "magma", 
                         aesthetics = "color", 
                         begin = 0.8, 
                         end = 0.001)
p.beta.its.mod <- p.beta.its +
  ggtitle('ITS2', subtitle = 'NMDS - Bray-Curtis dissimilarity') +
      scale_colour_viridis_d(option = "magma", 
                         aesthetics = "color", 
                         begin = 0.8, 
                         end = 0.001)
  
ggarrange(p.beta.16.mod, p.beta.its.mod)
```

```
ampits <- phyloseq_to_ampvis2(psits.f)
ampits
```

```
## ampvis2 object with 5 elements. 
## Summary of OTU table:
## [1]      35     334  114213     940    8389    2940 3263.23
## 
## Assigned taxonomy:
##     Kingdom      Phylum       Class       Order      Family       Genus 
##   334(100%)   334(100%)  160(47.9%) 143(42.81%) 139(41.62%) 130(38.92%) 
##     Species 
## 112(33.53%) 
## 
## Metadata variables: 5 
##  SampleID, Substrate, Repeat, Time, Day
```

```
ampvis2::amp_heatmap(ampits,
            tax_show = 40,
            group_by = "Day",
            facet_by = "Substrate",
            tax_aggregate = "Phylum",
            # tax_add = "Phylum",
            normalise=TRUE,
            showRemainingTaxa = TRUE)
```

```
p1 <- plot_alpha_w_toc_2(psits.f, group="Repeat", metric="Observed")
p2 <- plot_alpha_w_toc_2(psits.f, group="Repeat", metric="Shannon")
p3 <- plot_alpha_w_toc_2(psits.f, group="Repeat", metric="InvSimpson")

p_alpha <- ggpubr::ggarrange(p1, p2, p3, nrow = 1)
p_alpha
```

Only Ascomycota

```
ampits.nematode <- ampvis2::amp_filter_taxa(ampits, "Ascomycota" ,normalise = TRUE)
ampvis2::amp_heatmap(ampits.nematode,
            tax_show = 40,
            group_by = "Day",
            facet_by = "Substrate",
            tax_aggregate = "Genus",
            # tax_add = "Genus",
            normalise=FALSE,
            showRemainingTaxa = TRUE)
```

Only Nematoda

```
ampits.nematode <- ampvis2::amp_filter_taxa(ampits, "Nematoda" ,normalise = TRUE)
ampvis2::amp_heatmap(ampits.nematode,
            tax_show = 40,
            group_by = "Day",
            facet_by = "Substrate",
            tax_aggregate = "Species",
            tax_add = "Genus",
            normalise=FALSE,
            showRemainingTaxa = TRUE)
```

## 0.4 data normalisation and filtration

```
ps.ff <- phyloseq::filter_taxa(ps.f, function(x) sum(x > 5) > (0.1*length(x)), TRUE)

ps.f.a <- prune_samples(sample_data(ps.f)$Substrate %in% c('A'), ps.f) 
ps.f.a  <- prune_taxa(taxa_sums(ps.f.a) > 0, ps.f.a)   
ps.ff.a <- phyloseq::filter_taxa(ps.f.a, function(x) sum(x > 5) > (0.1*length(x)), TRUE)

ps.f.c <- prune_samples(sample_data(ps.f)$Substrate %in% c('C'), ps.f) 
ps.f.c  <- prune_taxa(taxa_sums(ps.f.c) > 0, ps.f.c)   
ps.ff.c <- phyloseq::filter_taxa(ps.f.c, function(x) sum(x > 5) > (0.1*length(x)), TRUE)

ps.f2.a <- phyloseq::filter_taxa(ps.f.a, function(x) sum(x > 5) > (0.1*length(x)), TRUE)
ps.f2.c <- phyloseq::filter_taxa(ps.f.c, function(x) sum(x > 5) > (0.1*length(x)), TRUE)
```

```
out.f2.a <-  ANCOMBC::ancombc2(data=ps.f2.a, 
  fix_formula = "Repeat",
  prv_cut = 0,
  p_adj_method = "BH",
  tax_level = NULL,
  rand_formula= NULL,
  pseudo = 0, 
  pseudo_sens = FALSE,
  s0_perc = 0.05,
  group = "Repeat",
  struc_zero = FALSE,
  neg_lb = TRUE,
  alpha = 0.05,
  n_cl = 20, 
  verbose = TRUE,
  global = FALSE, 
  pairwise = FALSE, 
  dunnet = FALSE, 
  trend = FALSE
  )

out.f2.c <-  ANCOMBC::ancombc2(data=ps.f2.c, 
  fix_formula = "Repeat",
  prv_cut = 0,
  p_adj_method = "BH",
  tax_level = NULL,
  rand_formula= NULL,
  pseudo = 0, 
  pseudo_sens = FALSE,
  s0_perc = 0.05,
  group = "Repeat",
  struc_zero = FALSE,
  neg_lb = TRUE,
  alpha = 0.05,
  n_cl = 20, 
  verbose = TRUE,
  global = FALSE, 
  pairwise = FALSE, 
  dunnet = FALSE, 
  trend = FALSE
  )
```

```
ps.f2.a.anc <- norm_anc(ps.f2.a, out.f2.a)
ps.f2.c.anc <- norm_anc(ps.f2.c, out.f2.c)

beta_custom_norm_NMDS_elli_w(ps.f2.a.anc, Color = "Repeat", Group = "Repeat")
```

```
## Run 0 stress 0.07429029 
## Run 1 stress 0.2315953 
## Run 2 stress 0.07429044 
## ... Procrustes: rmse 0.0002792688  max resid 0.0007346183 
## ... Similar to previous best
## Run 3 stress 0.07429038 
## ... Procrustes: rmse 0.0001203206  max resid 0.0003189117 
## ... Similar to previous best
## Run 4 stress 0.07429032 
## ... Procrustes: rmse 5.506845e-05  max resid 0.0001447092 
## ... Similar to previous best
## Run 5 stress 0.07429028 
## ... New best solution
## ... Procrustes: rmse 2.731641e-05  max resid 7.31658e-05 
## ... Similar to previous best
## Run 6 stress 0.07429036 
## ... Procrustes: rmse 0.0001277739  max resid 0.0003398777 
## ... Similar to previous best
## Run 7 stress 0.07429042 
## ... Procrustes: rmse 0.0002355085  max resid 0.0006176544 
## ... Similar to previous best
## Run 8 stress 0.07429032 
## ... Procrustes: rmse 0.0001482414  max resid 0.0003900916 
## ... Similar to previous best
## Run 9 stress 0.0742903 
## ... Procrustes: rmse 5.231897e-05  max resid 0.0001387494 
## ... Similar to previous best
## Run 10 stress 0.07429032 
## ... Procrustes: rmse 8.066035e-05  max resid 0.0002159956 
## ... Similar to previous best
## Run 11 stress 0.07429049 
## ... Procrustes: rmse 0.000193743  max resid 0.0005106779 
## ... Similar to previous best
## Run 12 stress 0.07429033 
## ... Procrustes: rmse 0.0001022038  max resid 0.0002721742 
## ... Similar to previous best
## Run 13 stress 0.07429033 
## ... Procrustes: rmse 3.477851e-05  max resid 9.256538e-05 
## ... Similar to previous best
## Run 14 stress 0.07429038 
## ... Procrustes: rmse 0.000142861  max resid 0.0003816703 
## ... Similar to previous best
## Run 15 stress 0.07429029 
## ... Procrustes: rmse 2.128628e-05  max resid 6.356351e-05 
## ... Similar to previous best
## Run 16 stress 0.07429052 
## ... Procrustes: rmse 0.0002847624  max resid 0.0007439491 
## ... Similar to previous best
## Run 17 stress 0.07429029 
## ... Procrustes: rmse 3.785926e-05  max resid 0.0001020905 
## ... Similar to previous best
## Run 18 stress 0.1171256 
## Run 19 stress 0.1171259 
## Run 20 stress 0.07429033 
## ... Procrustes: rmse 0.0001011432  max resid 0.0002689921 
## ... Similar to previous best
## *** Best solution repeated 14 times
```

```
beta_custom_norm_NMDS_elli_w(ps.f2.c.anc, Color = "Repeat", Group = "Repeat")
```

```
## Wisconsin double standardization
## Run 0 stress 0.1039491 
## Run 1 stress 0.1297154 
## Run 2 stress 0.1039492 
## ... Procrustes: rmse 6.624435e-05  max resid 0.0001588619 
## ... Similar to previous best
## Run 3 stress 0.1039491 
## ... New best solution
## ... Procrustes: rmse 1.364221e-05  max resid 3.340244e-05 
## ... Similar to previous best
## Run 4 stress 0.1039491 
## ... New best solution
## ... Procrustes: rmse 3.507746e-05  max resid 8.598372e-05 
## ... Similar to previous best
## Run 5 stress 0.1039492 
## ... Procrustes: rmse 3.631614e-05  max resid 0.000117302 
## ... Similar to previous best
## Run 6 stress 0.1039491 
## ... Procrustes: rmse 1.996358e-05  max resid 4.730391e-05 
## ... Similar to previous best
## Run 7 stress 0.1039491 
## ... New best solution
## ... Procrustes: rmse 4.003559e-06  max resid 1.208736e-05 
## ... Similar to previous best
## Run 8 stress 0.1782963 
## Run 9 stress 0.1039491 
## ... New best solution
## ... Procrustes: rmse 1.547874e-06  max resid 4.011174e-06 
## ... Similar to previous best
## Run 10 stress 0.1039491 
## ... Procrustes: rmse 1.038541e-05  max resid 2.804712e-05 
## ... Similar to previous best
## Run 11 stress 0.1039491 
## ... Procrustes: rmse 6.262294e-06  max resid 1.783688e-05 
## ... Similar to previous best
## Run 12 stress 0.1039491 
## ... Procrustes: rmse 3.22926e-05  max resid 7.795742e-05 
## ... Similar to previous best
## Run 13 stress 0.1039491 
## ... Procrustes: rmse 2.4952e-05  max resid 5.474128e-05 
## ... Similar to previous best
## Run 14 stress 0.1039491 
## ... Procrustes: rmse 4.012168e-05  max resid 9.280115e-05 
## ... Similar to previous best
## Run 15 stress 0.1891191 
## Run 16 stress 0.1039491 
## ... Procrustes: rmse 6.071329e-06  max resid 1.470288e-05 
## ... Similar to previous best
## Run 17 stress 0.1782881 
## Run 18 stress 0.1039491 
## ... Procrustes: rmse 7.529112e-06  max resid 1.749766e-05 
## ... Similar to previous best
## Run 19 stress 0.1039491 
## ... Procrustes: rmse 7.677729e-05  max resid 0.0001829159 
## ... Similar to previous best
## Run 20 stress 0.1039491 
## ... Procrustes: rmse 8.082714e-05  max resid 0.0001946092 
## ... Similar to previous best
## *** Best solution repeated 10 times
```

```
ps.f2.a.anc <- norm_anc(ps.f2.a, out.f2.a)
ps.f2.c.anc <- norm_anc(ps.f2.c, out.f2.c)
```

## 0.5 16S network analasys

```
netfull.a <- SpiecEasi::spiec.easi(ps.f2.a, method='mb', pulsar.params=list(rep.num=500))
netfull.c <- SpiecEasi::spiec.easi(ps.f2.c, method='mb', pulsar.params=list(rep.num=500))
```

```
igfull.a <- SpiecEasi::adj2igraph(getRefit(netfull.a), vertex.attr=list(name=taxa_names(ps.f2.a)))
igfull.c <- SpiecEasi::adj2igraph(getRefit(netfull.c), vertex.attr=list(name=taxa_names(ps.f2.c)))

modfull.a <- cluster_fast_greedy(igfull.a)
modfull.c <- cluster_fast_greedy(igfull.c)

ig.a.mod <- igfull.a
V(ig.a.mod)$color <-  modfull.a$membership

ig.c.mod <- igfull.c
V(ig.c.mod)$color <-  modfull.c$membership

print(modfull.a)
```

```
## IGRAPH clustering fast greedy, groups: 8, mod: 0.33
## + groups:
##   $`1`
##    [1] "Seq864"  "Seq176"  "Seq231"  "Seq424"  "Seq621"  "Seq146"  "Seq503" 
##    [8] "Seq843"  "Seq813"  "Seq804"  "Seq599"  "Seq403"  "Seq336"  "Seq45"  
##   [15] "Seq1461" "Seq691"  "Seq920"  "Seq14"   "Seq1054" "Seq1144" "Seq1192"
##   [22] "Seq1402" "Seq440"  "Seq1186" "Seq133"  "Seq263"  "Seq533"  "Seq1089"
##   [29] "Seq629"  "Seq1460" "Seq630"  "Seq923"  "Seq1015" "Seq769"  "Seq469" 
##   [36] "Seq778"  "Seq1356" "Seq768"  "Seq546"  "Seq467"  "Seq98"   "Seq885" 
##   [43] "Seq373"  "Seq639"  "Seq399"  "Seq1288" "Seq366"  "Seq386"  "Seq478" 
##   [50] "Seq383"  "Seq715"  "Seq280"  "Seq260"  "Seq408"  "Seq174"  "Seq278" 
##   [57] "Seq58"   "Seq389"  "Seq91"   "Seq1036" "Seq1247" "Seq298"  "Seq572" 
##   + ... omitted several groups/vertices
```

```
print(modfull.c)
```

```
## IGRAPH clustering fast greedy, groups: 9, mod: 0.41
## + groups:
##   $`1`
##    [1] "Seq89"   "Seq210"  "Seq693"  "Seq801"  "Seq175"  "Seq704"  "Seq454" 
##    [8] "Seq240"  "Seq108"  "Seq28"   "Seq423"  "Seq711"  "Seq1021" "Seq329" 
##   [15] "Seq675"  "Seq369"  "Seq1323" "Seq676"  "Seq344"  "Seq1200" "Seq171" 
##   [22] "Seq174"  "Seq432"  "Seq839"  "Seq58"   "Seq148"  "Seq948"  "Seq172" 
##   [29] "Seq224"  "Seq300"  "Seq314"  "Seq809"  "Seq1394" "Seq712"  "Seq192" 
##   [36] "Seq927"  "Seq251"  "Seq505"  "Seq99"   "Seq651" 
##   
##   $`2`
##    [1] "Seq21"   "Seq212"  "Seq30"   "Seq698"  "Seq55"   "Seq264"  "Seq323" 
##   + ... omitted several groups/vertices
```

```
wtc.ig.a.mod <- cluster_walktrap(ig.a.mod)
wtc.ig.c.mod <- cluster_walktrap(ig.c.mod)

modularity(wtc.ig.a.mod)
```

```
## [1] 0.2822789
```

```
modularity(wtc.ig.c.mod)
```

```
## [1] 0.3299702
```

7th group from the chernozem

```
groups.c <- data.frame('ID' = modfull.c$names, 'Group' = modfull.c$membership)
groups.a <- data.frame('ID' = modfull.a$names, 'Group' = modfull.a$membership)

id.7 <- ps.f2.c@tax_table %>% 
  data.frame() %>% 
  rownames_to_column('ID') %>% 
  right_join(groups.c) %>% 
  filter(Group %in% c('7')) %>% 
pull('ID')

amp.ff.c <- phyloseq_to_ampvis2(ps.f2.c)
amp.ff.c.7 <- ampvis2::amp_filter_taxa(amp.ff.c, tax_vector = id.7, normalise = TRUE)


ampvis2::amp_heatmap(amp.ff.c.7,
            tax_show = 30,
            tax_aggregate = "OTU",
            tax_add = "Genus",
            group_by = "Day",
            plot_values_size = 4,
            showRemainingTaxa = TRUE, normalise = FALSE)
```

### 0.5.1 creating net`s plots and treeplots

```
library(GGally)

set.seed(1516)

pl_pal <-  viridis::magma(n = 9) 
# option = "magma", aesthetics = "color", begin = 0.8, end = 0.001

p.net.a <- ggnet2(ig.a.mod, 
        size ="0.7",
       node.color = "color", 
       label = FALSE, 
       node.size = 2, 
       label.size = 1,
       mode = "kamadakawai") + 
  guides(color=guide_legend(title="color"), size = FALSE)  + 
  scale_color_manual(values = pl_pal) +
    theme(legend.position="none")  + 
  ggtitle('A', subtitle = 'modularity - 0.28')

p.net.c <- ggnet2(ig.c.mod, 
      size ="0.7",
       node.color = "color", 
       label = FALSE, 
      node.size = 2, 
       label.size = 1,
       mode = "kamadakawai") + 
  guides(color=guide_legend(title="Groups"), size = FALSE)  + 
  scale_color_manual(values = pl_pal)  +
    theme(legend.position="none")  + 
  ggtitle('C', subtitle = 'modularity - 0.33')

ggarrange(p.net.a, p.net.c)
```

Groups taxonomy for A

```
tx <- left_join(groups.a, ps.f2.a.anc@tax_table@.Data %>% data.frame() %>% rownames_to_column('ID')) %>% 
  add_column(abnd = taxa_sums(ps.f2.a.anc))


tx %>% 
  mutate(Phylum = case_when(Phylum %in% "Proteobacteria" ~ Class,
                            !Phylum %in% "Proteobacteria" ~ Phylum) %>% as.factor()) %>% 
  select(c("Genus", "Phylum", "Group", "abnd")) %>% 
  mutate(Group = as.factor(Group)) %>% 
  group_by(Genus, Phylum, Group) %>%
  mutate(Genus = str_replace_na(Genus, replacement = ' ') %>% as.factor()) %>% 
  summarise(area = sum(abnd)) %>% 
  mutate_if(is.character, as.factor) %>% 
  drop_na() %>% 
  filter(Group %in% c('1', '2', '4', '8')) %>% 
  ggplot(aes(area = area, 
             subgroup = Phylum, 
             label = Genus,
             fill = Group)) +
  treemapify::geom_treemap() +
  treemapify::geom_treemap_subgroup_border(color = "white") +
  treemapify::geom_treemap_subgroup_text(place = "centre", 
                                         grow = T, 
                                         alpha = 0.7, 
                                         colour = "black", 
                                         fontface = "italic", 
                                         min.size = 2) +
  treemapify::geom_treemap_text(colour = "white", 
                                place = "topleft", 
                                grow = T, 
                                reflow = T, 
                                layout = 'squarified',
                                min.size = 4) +
  facet_wrap(~Group, labeller = labeller(Group = 
    c("1" = "Group1, rel=9.16%, ASVs=84",
      "2" = "Group2, rel=30.97%, ASVs=164",
      "4" = "Group4, rel=29.17%, ASVs=141",
      "8" = "Group8, rel=25.06%, ASVs=160"))) +
  scale_colour_viridis_d(option = "plasma", 
                         aesthetics = "fill", 
                         begin = 0.2, 
                         end = 0.7) +
  theme(legend.position="bottom")
```

Groups taxonomy for C

```
tx <- left_join(groups.c, ps.f2.c.anc@tax_table@.Data %>% data.frame() %>% rownames_to_column('ID')) %>% 
  add_column(abnd = taxa_sums(ps.f2.c.anc))

tx %>% 
  mutate(Phylum = case_when(Phylum %in% "Proteobacteria" ~ Class,
                            !Phylum %in% "Proteobacteria" ~ Phylum) %>% as.factor()) %>% 
  select(c("Genus", "Phylum", "Group", "abnd")) %>% 
  mutate(Group = as.factor(Group)) %>% 
  group_by(Genus, Phylum, Group) %>%
  mutate(Genus = str_replace_na(Genus, replacement = ' ') %>% as.factor()) %>% 
  summarise(area = sum(abnd)) %>% 
  mutate_if(is.character, as.factor) %>% 
  drop_na() %>% 
  filter(Group %in% c('1', '2', '4', '6','7','9')) %>% 
  ggplot(aes(area = area, 
             subgroup = Phylum, 
             label = Genus,
             fill = Group)) +
  treemapify::geom_treemap() +
  treemapify::geom_treemap_subgroup_border(color = "white") +
  treemapify::geom_treemap_subgroup_text(place = "centre", 
                                         grow = T, 
                                         alpha = 0.7, 
                                         colour = "black", 
                                         fontface = "italic", 
                                         min.size = 2) +
  treemapify::geom_treemap_text(colour = "white", 
                                place = "topleft", 
                                grow = T, 
                                reflow = T, 
                                layout = 'squarified',
                                min.size = 4) +
  facet_wrap(~Group, labeller = labeller(Group = 
    c("1" = "Group1, rel=8.44%, ASVs=40",
      "2" = "Group2, rel=21.35%, ASVs=58",
      "4" = "Group4, rel=15.66%, ASVs=76",
      "6" = "Group6, rel=10.94%, ASVs=26",
      "7" = "Group7, rel=18.25%, ASVs=55",
      "9" = "Group9, rel=19.66%, ASVs=68" ))) +
  scale_colour_viridis_d(option = "plasma", 
                         aesthetics = "fill", 
                         begin = 0.2, 
                         end = 0.7) +
  theme(legend.position="bottom")
```

### 0.5.2 adding aspiration plots

```
ps.f.r <- rarefy_even_depth(ps.f, rngseed = 1433) 
er <- estimate_richness(ps.f.r)

p.alpha.16 <- estimate_richness(ps.f.r) %>% 
  rownames_to_column("ID") %>% 
  mutate(ID = str_sub(ID, 2)) %>% 
  left_join(ps.f.r@sam_data %>%  data.frame() %>%  rownames_to_column("ID") %>% mutate(Repeat = as.factor(Repeat))) %>% 
  group_by(Substrate) %>% 
  pivot_longer(c( "Observed", "Shannon", "InvSimpson", "Respiration")) %>% 
  mutate(Index = factor(name, levels = c("Observed", "Shannon", "InvSimpson", "Respiration"))) %>% 
  ggplot(aes(y = value, x = Day, group = Substrate)) +
    # geom_line(aes(color = name),
    #         alpha = 1) +
    stat_summary(aes(y = value, color = Substrate), fun.y=mean, geom="line") +
    labs(x = "day",
       y = "index value") +  
  labs(title = "16S") + 
  geom_point(aes(shape = Index, color = Substrate)) +
  facet_wrap(~ Index, nrow = 2, scales = "free") +
    scale_colour_viridis_d(option = "magma", 
                         aesthetics = "color", 
                         begin = 0.8, 
                         end = 0.001) +
  theme_bw()

psits.f.r <- rarefy_even_depth(psits.f, rngseed = 1433) 
er.its <- estimate_richness(ps.f.r)

p.alpha.its <- estimate_richness(psits.f.r) %>% 
  rownames_to_column("ID") %>% 
  mutate(ID = str_sub(ID, 1)) %>% 
  left_join(psits.f.r@sam_data %>%  data.frame() %>%  rownames_to_column("ID") %>% mutate(Repeat = as.factor(Repeat))) %>% 
  left_join(ps.f.r@sam_data %>%  data.frame() %>%  rownames_to_column("ID") %>% dplyr::select(c("Repeat", "Respiration")) %>% mutate(Repeat = as.factor(Repeat))) %>% 
  group_by(Substrate) %>% 
  pivot_longer(c( "Observed", "Shannon", "InvSimpson", "Respiration")) %>% 
  mutate(Index = factor(name, levels = c("Observed", "Shannon", "InvSimpson", "Respiration"))) %>% 
  mutate(Substrate = as.character(Substrate)) %>%
  ggplot(aes(y = value, x = Day, group = Substrate)) +
    stat_summary(aes(y = value, color = Substrate), fun.y=mean, geom="line") +
    labs(x = "day",
       y = "index value") +
  labs(title = "ITS2") + 
  geom_point(aes(color = Substrate, shape = Index)) +
  facet_wrap(~ Index, nrow = 2, scales = "free") +
    scale_colour_viridis_d(option = "magma", 
                         aesthetics = "color", 
                         begin = 0.8, 
                         end = 0.001) +
  theme_bw() +
  theme(legend.position = "bottom")

g_legend<-function(a.gplot){
  tmp <- ggplot_gtable(ggplot_build(a.gplot))
  leg <- which(sapply(tmp$grobs, function(x) x$name) == "guide-box")
  legend <- tmp$grobs[[leg]]
  return(legend)}

mylegend <- g_legend(p.alpha.its)


gridExtra::grid.arrange(gridExtra::arrangeGrob(p.alpha.16 + theme(legend.position="none"),
                         p.alpha.its + theme(legend.position="none"),
                         nrow=1),
             mylegend, nrow=2,heights=c(12, 1))
```

### 0.5.3 formal stats

```
dist <- phyloseq::distance(ps.f, "euclidean")
metadata <- as(sample_data(ps.f@sam_data), "data.frame")
vegan::adonis2(dist ~ Substrate + Time, data = metadata)
```

```
## Permutation test for adonis under reduced model
## Terms added sequentially (first to last)
## Permutation: free
## Number of permutations: 999
## 
## vegan::adonis2(formula = dist ~ Substrate + Time, data = metadata)
##           Df  SumOfSqs      R2      F Pr(>F)    
## Substrate  1  67513024 0.06872 2.5890  0.001 ***
## Time       1  80460267 0.08190 3.0855  0.001 ***
## Residual  32 834473651 0.84938                  
## Total     34 982446943 1.00000                  
## ---
## Signif. codes:  0 '***' 0.001 '**' 0.01 '*' 0.05 '.' 0.1 ' ' 1
```

```
dist <- phyloseq::distance(ps.f, "euclidean")
metadata <- as(sample_data(ps.f@sam_data), "data.frame")
vegan::adonis2(dist ~ Substrate + Time, data = metadata)
```

```
## Permutation test for adonis under reduced model
## Terms added sequentially (first to last)
## Permutation: free
## Number of permutations: 999
## 
## vegan::adonis2(formula = dist ~ Substrate + Time, data = metadata)
##           Df  SumOfSqs      R2      F Pr(>F)    
## Substrate  1  67513024 0.06872 2.5890  0.001 ***
## Time       1  80460267 0.08190 3.0855  0.001 ***
## Residual  32 834473651 0.84938                  
## Total     34 982446943 1.00000                  
## ---
## Signif. codes:  0 '***' 0.001 '**' 0.01 '*' 0.05 '.' 0.1 ' ' 1
```

```
dist <- phyloseq::distance(psits.f, "euclidean")
metadata <- as(sample_data(psits.f@sam_data), "data.frame")
vegan::adonis2(dist ~ Substrate + Time, data = metadata)
```

```
## Permutation test for adonis under reduced model
## Terms added sequentially (first to last)
## Permutation: free
## Number of permutations: 999
## 
## vegan::adonis2(formula = dist ~ Substrate + Time, data = metadata)
##           Df  SumOfSqs      R2      F Pr(>F)    
## Substrate  1  11323364 0.07179 2.6578  0.001 ***
## Time       1  10074181 0.06387 2.3646  0.006 ** 
## Residual  32 136333195 0.86434                  
## Total     34 157730740 1.00000                  
## ---
## Signif. codes:  0 '***' 0.001 '**' 0.01 '*' 0.05 '.' 0.1 ' ' 1
```

```
sessionInfo()
```

```
## R version 4.3.2 (2023-10-31)
## Platform: x86_64-pc-linux-gnu (64-bit)
## Running under: Ubuntu 20.04.6 LTS
## 
## Matrix products: default
## BLAS:   /usr/lib/x86_64-linux-gnu/blas/libblas.so.3.9.0 
## LAPACK: /usr/lib/x86_64-linux-gnu/lapack/liblapack.so.3.9.0
## 
## locale:
##  [1] LC_CTYPE=en_US.UTF-8       LC_NUMERIC=C              
##  [3] LC_TIME=en_US.UTF-8        LC_COLLATE=en_US.UTF-8    
##  [5] LC_MONETARY=en_US.UTF-8    LC_MESSAGES=en_US.UTF-8   
##  [7] LC_PAPER=en_US.UTF-8       LC_NAME=C                 
##  [9] LC_ADDRESS=C               LC_TELEPHONE=C            
## [11] LC_MEASUREMENT=en_US.UTF-8 LC_IDENTIFICATION=C       
## 
## time zone: Europe/Moscow
## tzcode source: system (glibc)
## 
## attached base packages:
## [1] stats     graphics  grDevices utils     datasets  methods   base     
## 
## other attached packages:
##  [1] GGally_2.2.1    ggpubr_0.6.0    ggforce_0.4.2   ggrepel_0.9.5  
##  [5] SpiecEasi_1.1.3 igraph_2.0.2    phyloseq_1.44.0 lubridate_1.9.3
##  [9] forcats_1.0.0   stringr_1.5.1   dplyr_1.1.4     purrr_1.0.2    
## [13] readr_2.1.5     tidyr_1.3.1     tibble_3.2.1    ggplot2_3.5.0  
## [17] tidyverse_2.0.0
## 
## loaded via a namespace (and not attached):
##   [1] bitops_1.0-7            gridExtra_2.3           permute_0.9-7          
##   [4] rlang_1.1.3             magrittr_2.0.3          ade4_1.7-22            
##   [7] compiler_4.3.2          mgcv_1.9-1              systemfonts_1.0.6      
##  [10] vctrs_0.6.5             reshape2_1.4.4          pkgconfig_2.0.3        
##  [13] shape_1.4.6.1           crayon_1.5.2            fastmap_1.1.1          
##  [16] backports_1.4.1         XVector_0.40.0          labeling_0.4.3         
##  [19] utf8_1.2.4              rmarkdown_2.26          ggfittext_0.10.2       
##  [22] tzdb_0.4.0              network_1.18.2          xfun_0.42              
##  [25] glmnet_4.1-8            zlibbioc_1.46.0         cachem_1.0.8           
##  [28] GenomeInfoDb_1.36.4     jsonlite_1.8.8          biomformat_1.28.0      
##  [31] highr_0.10              rhdf5filters_1.12.1     Rhdf5lib_1.22.1        
##  [34] tweenr_2.0.3            huge_1.3.5              VGAM_1.1-10            
##  [37] broom_1.0.5             parallel_4.3.2          cluster_2.1.6          
##  [40] R6_2.5.1                RColorBrewer_1.1-3      bslib_0.6.1            
##  [43] stringi_1.8.3           car_3.1-2               jquerylib_0.1.4        
##  [46] Rcpp_1.0.12             iterators_1.0.14        knitr_1.45             
##  [49] IRanges_2.34.1          Matrix_1.6-5            splines_4.3.2          
##  [52] timechange_0.3.0        tidyselect_1.2.1        viridis_0.6.5          
##  [55] rstudioapi_0.15.0       abind_1.4-5             yaml_2.3.8             
##  [58] vegan_2.6-4             codetools_0.2-19        lattice_0.22-5         
##  [61] plyr_1.8.9              Biobase_2.60.0          withr_3.0.0            
##  [64] coda_0.19-4.1           evaluate_0.23           survival_3.5-8         
##  [67] ggstats_0.5.1           polyclip_1.10-6         Biostrings_2.68.1      
##  [70] pillar_1.9.0            carData_3.0-5           foreach_1.5.2          
##  [73] stats4_4.3.2            plotly_4.10.4           generics_0.1.3         
##  [76] RCurl_1.98-1.14         S4Vectors_0.38.2        hms_1.1.3              
##  [79] munsell_0.5.0           scales_1.3.0            glue_1.7.0             
##  [82] pulsar_0.3.11           lazyeval_0.2.2          tools_4.3.2            
##  [85] data.table_1.15.2       ggsignif_0.6.4          cowplot_1.1.3          
##  [88] rhdf5_2.44.0            grid_4.3.2              ape_5.7-1              
##  [91] colorspace_2.1-0        nlme_3.1-164            GenomeInfoDbData_1.2.10
##  [94] cli_3.6.2               intergraph_2.0-4        ampvis2_2.7.28         
##  [97] fansi_1.0.6             viridisLite_0.4.2       gtable_0.3.4           
## [100] rstatix_0.7.2           sass_0.4.8              digest_0.6.35          
## [103] BiocGenerics_0.46.0     sna_2.7-2               htmlwidgets_1.6.4      
## [106] farver_2.1.1            htmltools_0.5.7         multtest_2.56.0        
## [109] lifecycle_1.0.4         httr_1.4.7              statnet.common_4.9.0   
## [112] treemapify_2.5.6        MASS_7.3-60.0.1
```
