# Supplementary material for: Dynamics of Cellulose Degradation by Soil Microorganisms from Two Contrasting Soil Types
Source: Microorganisms. 2024 Aug 21;12(8):1728. doi: 10.3390/microorganisms12081728 (PMC11356995; doi:10.3390/microorganisms12081728)
Supplement: Supplementary file 1 [file microorganisms-12-01728-s001.zip › microorganisms-3132235-supplementary/Supplement_2.pdf]

Table S1. The main physical and agrochemical characteristics of the analyzed soil samples *[10]*

**Parameter**	**Units**	**Soil type**	
		Sod-podzolic	Chernozem
**Particle size (mm) distribution**			
**1-0.25**	%	15.1	1.8
**0.25-0.10**	%	16.4	1.1
**0.10-0.05**	%	23.26	10.86
**0.05-0.01**	%	28.64	33.36
**0.01-0.005**	%	1.8	7.68
**0.005-0.001**	%	3.2	11.08
**<0.001**	%	11.6	34.12
**Agro-chemical analysis**			
**pH**	pH units	6.05	7.32
**N**	%	0.22	0.38
**P**	mg/kg	85	121
**K**	mg/kg	60	155
**C**	%	2.48	8.75
**Ca**	mmol/100 g	3.25	30.62
**Mg**	mmol/100 g	2.45	3.82
